# Adjunctive Antimicrobial Photodynamic Therapy in Non-Surgical Periodontal Treatment: Clinical and Microbiological Insights from a Randomized Split-Mouth Trial

**DOI:** 10.3390/healthcare14162459

**Published:** 2026-08-09

**Authors:** Alessia Pardo, Gabriel Gallo, Annarita Signoriello, Elena Messina, Gloria Burlacchini, Caterina Signoretto, Giorgio Lombardo, Massimo Albanese

**Affiliations:** 1 Dentistry and Maxillo-Facial Surgery Unit, Department of Surgery, Dentistry, Pediatrics and Gynecology (DIPSCOMI), University of Verona, Piazzale L.A. Scuro 10, 37134 Verona, Italy; alessia.pardo@univr.it (A.P.); gabriel.gallo@studenti.univr.it (G.G.); giorgio.lombardo@univr.it (G.L.); massimo.albanese@univr.it (M.A.); 2 Diagnostic and Public Health Department, University of Verona, 37134 Verona, Italy; gloria.burlacchini@univr.it (G.B.); caterina.signoretto@univr.it (C.S.)

**Keywords:** antimicrobial photodynamic therapy (aPDT), periodontitis, scaling and root planing (SRP), non-surgical periodontal therapy, randomized clinical trial, periodontal inflammation

## Abstract

**Background/Objectives:** Antimicrobial photodynamic therapy (aPDT) has been proposed as an adjunct to non-surgical periodontal therapy, although its benefits remain debated. This study evaluated the clinical and microbiological effects of aPDT combined with ultrasonic scaling and root planing (US-SRP) compared with US-SRP alone in patients with periodontitis. **Methods:** In this randomized split-mouth clinical trial, 40 patients with stage II–III periodontitis were included. Two non-adjacent sites with probing pocket depth (PPD) ≥ 5 mm were assigned to either control (US-SRP) or test (aPDT + US-SRP) treatment. Clinical parameters, including PPD, bleeding on probing (BOP), plaque index (PI), gingival recession (REC), and clinical attachment level (CAL), were assessed at baseline and after 90 days. Subgingival plaque samples were analyzed by multiplex PCR for major periodontal pathogens. **Results:** No statistically significant differences were detected for microbiological outcomes at 90 days between groups. Significant greater variations were observed at T3 in the aPDT group for PPD (*p* = 0.04), BOP (*p* = 0.03) and PI (*p* = 0.005), whereas the control group showed a significant reduction only for PI (*p* = 0.01). Regarding comparisons of Δ(T0–T3) between the aPDT group and the control group, the paired t-test was significant for PPD, REC and CAL (*p* < 0.001), with a Cohen’s d_av_ < 0.5 for all three parameters (small effect sizes). In addition, the aPDT group showed a statistically significant reduction in BOP (*p* = 0.03 *) from T0 (92%) to T3 (50%). Exploratory analyses confirmed the same trend for BOP reduction in the aPDT group both in smokers and non-smokers. **Conclusions:** Despite the fact that adjunctive aPDT seems to offer predictable clinical outcomes in terms of PPD and BOP reductions, further larger and stratified studies are needed to verify if this treatment can provide superior benefits compared with conventional non-surgical periodontal therapy.

## 1. Introduction

Periodontal disease is a highly prevalent condition, affecting approximately 20–50% of the global population, and represents a major public health concern [1]. Periodontitis is a chronic inflammatory disease associated with a dysbiotic bacterial biofilm and characterized by the progressive destruction of the tooth-supporting tissues, including the cementum, periodontal ligament, and alveolar bone. Clinically, it is characterized by increased probing pocket depth, clinical attachment loss, and bleeding on probing. Gingival recession, tooth mobility, and halitosis may also occur [2].

The definitions and diagnostic criteria used to identify periodontitis have varied considerably over time [3]. According to the current classification system, periodontitis is categorized into stages and grades based on disease severity, treatment complexity, and the estimated rate of progression [4]. The disease is initiated by the accumulation of a dysbiotic oral biofilm, which triggers a host-mediated inflammatory response and ultimately leads to connective tissue attachment loss and alveolar bone resorption [5,6]. However, individual susceptibility to disease onset and progression varies considerably and is influenced by environmental, behavioural, and systemic risk factors [7].

Several adjunctive approaches have been investigated to improve the outcomes of non-surgical periodontal treatment, including antimicrobial photodynamic therapy [8]. Nevertheless, the primary goal of periodontal therapy remains the disruption and removal of supra- and subgingival biofilm and calculus through non-surgical mechanical debridement. Scaling and root planing (SRP) is considered the cornerstone of non-surgical periodontal treatment [9,10]. However, the complete elimination of periodontal pathogens may be difficult to achieve, particularly in deep periodontal pockets and anatomically complex sites, where access for mechanical instrumentation is limited and residual biofilm may persist [11,12].

To overcome these limitations, several adjunctive strategies have been proposed, including locally or systemically administered antimicrobial agents, air-polishing systems, and other supportive therapies [13,14]. However, concerns regarding antimicrobial resistance and the potential adverse effects associated with conventional antimicrobial agents have encouraged the development of alternative treatment strategies.

Antimicrobial photodynamic therapy (aPDT) has emerged as a potentially useful adjunct to non-surgical periodontal treatment. This technique involves the application of a photosensitizing agent followed by irradiation with light of an appropriate wavelength. In the presence of oxygen, photoactivation generates reactive oxygen species capable of damaging bacterial membranes, proteins, and nucleic acids. The predominantly local effect of aPDT, together with its low potential to promote microbial resistance, makes it an attractive alternative to conventional adjunctive antimicrobial therapies [15,16,17].

Nevertheless, the additional clinical benefit of aPDT remains uncertain. Previous randomized clinical trials have reported inconsistent findings. Some studies have demonstrated additional improvements in probing pocket depth, clinical attachment level, bleeding indices, or subgingival microbial composition when aPDT was combined with SRP, whereas others have reported limited or no clinically relevant benefit compared with mechanical treatment alone [18,19,20,21,22]. For example, previous evidence has suggested that laser-assisted periodontal therapy may influence wound healing and inflammatory outcomes, although the magnitude of its additional clinical benefit remains uncertain [18]. Conversely, a recent split-mouth randomized clinical trial reported greater short-term probing pocket depth reduction and measurable changes in the subgingival microbial composition following repeated aPDT applications [23].

The findings of systematic reviews and meta-analyses are similarly heterogeneous. Some quantitative syntheses have reported modest short-term improvements in probing pocket depth and clinical attachment level following adjunctive aPDT, particularly at approximately 3 months. However, the magnitude and statistical significance of these benefits have not consistently persisted during longer follow-up periods [24,25,26,27]. Furthermore, evidence specifically concerning smokers suggests that adjunctive aPDT may not provide consistent improvements in probing pocket depth or clinical attachment gain in this population [28].

This variability may partly be explained by the considerable methodological heterogeneity among available studies, including differences in photosensitizing agents, light sources, wavelengths, energy parameters, irradiation times, treatment frequency, timing of application, baseline pocket depth, disease severity, smoking status, follow-up duration, and microbiological assessment methods. In particular, single and repeated aPDT applications should not necessarily be regarded as equivalent, and the clinical relevance of repeated treatment protocols remains incompletely established [23,29].

In addition, many previous studies have focused primarily on overall clinical outcomes, whereas fewer trials have simultaneously evaluated clinical and microbiological responses or explored whether treatment effects differ according to patient- and site-related characteristics. Tooth anatomy may influence the effectiveness of mechanical instrumentation because access to root surfaces and periodontal pockets is more difficult in anatomically complex or multi-rooted teeth. Similarly, smoking may alter the inflammatory response and periodontal healing and may therefore influence the apparent benefit of an adjunctive antimicrobial intervention.

Consequently, an important knowledge gap remains regarding whether repeated adjunctive aPDT provides measurable clinical and microbiological benefits beyond those obtained with ultrasonic scaling and root planing under standardized treatment conditions. It also remains unclear whether the response to treatment is influenced by smoking status or tooth type. A split-mouth design may be particularly suitable for addressing these questions because each participant serves as their own control, thereby reducing the influence of inter-individual biological and behavioural variability.

The present study therefore investigated the effects of repeated adjunctive aPDT administered according to a standardized three-session protocol. The primary aim of this randomized split-mouth clinical trial was to compare the clinical effectiveness of repeated aPDT combined with ultrasonic scaling and root planing with that of ultrasonic scaling and root planing alone in patients with stage II–III periodontitis. The secondary aim was to assess qualitative changes in selected periodontal pathogens. Exploratory analyses were also performed to investigate whether the clinical response differed according to smoking status and tooth type.

We hypothesized that adjunctive aPDT would result in greater reductions in probing pocket depth and inflammatory parameters than ultrasonic scaling and root planing alone.

## 2. Materials and Methods

### 2.1. Trial Design and Setting

This randomized, split-mouth, controlled, clinical trial was conducted at the Periodontology Unit, Department of Dentistry and Maxillofacial Surgery, University of Verona, Italy.

#### 2.1.1. Ethics Approval, Human Ethics and Consent to Participate Declarations

The study protocol was approved by the local Verona Ethics Committee (protocol code Prog. 4242CESC, 3 January 2024).

The study was performed in accordance with the Declaration of Helsinki and reported following CONSORT guidelines.

All human participants provided written informed consent prior to inclusion.

#### 2.1.2. Clinical Trial Registration

The study was registered at the registry ClinicalTrials.gov [Clinical trial number NCT07080762, date 9 July 2025 (this is a retrospective clinical registration: at the time of submission of the study protocol, the Ethics Committee did not require registration for feasibility or proof of concept studies. The study was registered in preparation for a manuscript for publication of the data)].

### 2.2. Study Population and Eligibility Criteria

Patients aged 18 years or older were recruited from an operator involved in the clinical trial. Patients were eligible if they had a diagnosis of periodontitis within the new classification of periodontal and peri-implant diseases and if they had not undergone any other non-surgical periodontal treatment in the year preceding enrolment. Diagnostic criteria outlined in the European Federation of Periodontology (EFP) guidelines were used [30].

### 2.3. Inclusion and Exclusion Criteria

Patients included in the study presented: age ≥ 18 years, diagnosis of stage II–III periodontitis according to EFP criteria [30], presence of at least 16 teeth, and at least two non-adjacent sites in different quadrants with PPD ≥ 5 mm. Patients who had taken periodontal treatment within the previous 6 months, antibiotic therapy within the previous 6 months, or had systemic conditions contraindicating periodontal therapy or aPDT, pregnancy, or breastfeeding were all excluded.

### 2.4. Randomization, Site-Matching, Allocation Concealment, and Blinding

A split-mouth design was adopted. For each participant, two eligible non-adjacent periodontal sites located in different quadrants were selected before treatment allocation. Site selection was matched according to tooth type and baseline probing pocket depth. The paired sites were required to be comparable in terms of tooth type, and the difference in baseline PPD between the two sites was not allowed to exceed 2 mm.

After both sites had been selected and baseline clinical and microbiological assessments had been completed, one site was randomly assigned to the test group (aPDT + US-SRP) and the other to the control group (US-SRP alone), using a computer-generated randomization sequence with a 1:1 allocation ratio. Allocation concealment was maintained using sequentially numbered, opaque, sealed envelopes, which were opened only after completion of baseline assessments.

The examiner performing the clinical measurements, the microbiologist, the statistician, and the participants were blinded to treatment allocation. The clinician administering aPDT could not be blinded because of the nature of the intervention.

### 2.5. Periodontal Treatment Protocol

All patients received full-mouth ultrasonic scaling and root planing (US-SRP) performed in three sessions at baseline (T0), 7 days (T1), and 14 days (T2).

In the test group, the HELBO^®^ antimicrobial photodynamic therapy system was used. An aqueous phenothiazinium-based photosensitizer [HELBO^®^ Biofilm Marker (bredent medical GmbH & Co.KG, Senden, Germany); 0.5 mL applicator; phenothiazin-5-ium, 3,7-bis(dimethylamino), chloride] was introduced into the periodontal pocket until the affected area was completely covered and was left in place for 3 min. After removal of the excess photosensitizer, the site was uniformly irradiated using a 660 nm diode laser with a power output of 100 mW in continuous-wave mode. Irradiation was performed for at least 1 min per cm^2^, corresponding to approximately 30 s per treated area and a total irradiation time of 60 s per site. The total energy delivered per site was 6 J. The procedure was repeated immediately after each US-SRP session at T0, T1, and T2. Patients received standardized oral hygiene instructions at baseline and during follow-up visits. The use of adjunctive antiseptic mouthwashes or systemic antibiotics was not allowed during the study period.

Patient compliance was monitored at each scheduled visit by recording attendance at the three treatment sessions and follow-up appointments. Participants were also asked whether they had used systemic antibiotics, antiseptic mouthwashes, or received any additional periodontal treatment during the study period. Adherence to the prescribed oral hygiene instructions was reinforced and assessed verbally at each visit. Any missed visit or protocol deviation was recorded in the study documentation.

### 2.6. Clinical Outcomes

Clinical parameters were recorded at baseline (T0) and after 90 days (T3), using a calibrated UNC-15 periodontal probe (Hu-Friedy Mfg. Co., LLC, Chicago, IL, USA): probing pocket depth (PPD), bleeding on probing (BOP), plaque index (PI), gingival recession (REC), clinical attachment level (CAL).

Measurements were performed at six sites per tooth by a single calibrated examiner blinded to treatment allocation and not involved in the treatment. As previously described [31], this examiner recorded that all the parameters were calibrated for adequate intra- and inter-examiner levels of reproducibility before the start of the study. The calibration for intra-examiner reproducibility was performed with double recording of 10 pockets for PPD, REC, and CAL in mm, and for BOP and PI in related scores, with an interval of 24 h between the first and second recording. Basic parameters of PPD (in mm) and PI (in score) were measured for 4 pockets utilized for this purpose: average values lower than 0.1 mm for PPD and 0.5 for PI were considered reliable as threshold limits from a clinical point of view. For inter-examiner reproducibility, the above-mentioned exercise was repeated by another dentist not involved in the treatments, according to the same method.

### 2.7. Microbiological Analysis

Subgingival plaque samples were collected from the same selected test and control sites at baseline (T0), 14 days (T2), and 90 days (T3). All samples were obtained by the same trained operator using a standardized procedure to ensure reproducibility across patients and study visits.

Before sampling, the selected site was isolated to minimize salivary contamination, and supragingival plaque was carefully removed without disturbing the subgingival biofilm. A sterile paper point was inserted into the periodontal pocket to the clinically accessible depth and maintained in position for [sampling duration]. Each paper point was immediately transferred into a sterile, individually labelled collection tube and stored at −4 °C until laboratory processing. The same sampling duration, collection materials, storage conditions, and transport procedures were used at all evaluation time points.

DNA from the plaque sampling was extracted using the GenElute™ commercial kit (Sigma-Aldrich, St. Louis, MO, USA), according to the manufacturer’s instructions. DNA concentration and purity were assessed using NanoDrop Microvolume Spectrophotometers (Thermo Fisher Scientific, MA, USA). Extracted DNA was stored at −20 °C until further use. Two different multiplex polymerase chain reaction (mPCR) assay were performed to qualitatively detect *Aggregatibacter actinomycetemcomitans*, *Porphyromonas gingivalis*, *Prevotella intermedia* (m1PCR), *Tannerella forsythia* and *Actinomyces naeslundii*. (m2PCR) [32]. These microorganisms were selected because they are commonly associated with periodontal dysbiosis and disease progression and could be simultaneously detected using the validated multiplex assay available in our laboratory. The first multiplex PCR (m1PCR) used a universal forward primer and a species-specific reverse primer to detect the different microorganisms detected. The second multiplex PCR (m2PCR) used primer pairs specific to each pathogen. PCR reactions were performed using the 5Prime Hot Master Mix (Quantabio, Beverly, MA, USA) according to the manufacturer’s instructions. Briefly, PCR reactions mixture were composed of 8 μL of 5 PRIME HotMaster Mix (2.5×), 100 nM of each primer, 50 ng of template, and Ultrapure DNase/RNase-free distilled water (Thermo Fisher Scientific, Waltham, MA, USA) to reach a final volume of 20 μL.

The primer sequences, expected amplicon sizes, and relevant references are reported in Table 1.

Each amplification run included a positive control containing reference DNA for the target microorganisms, a negative extraction control, and a no-template control to identify possible contamination. Samples were considered positive when an amplification product of the expected size was detected using electrophoresis migration. The analytical detection threshold of the assay was between 1 and 1.5 pg/µL of microbial DNA, while analytical specificity was previously verified using DNA from ATCC reference strains as the positive control. Laboratory analyses were performed by a microbiologist blinded to treatment allocation.

As the microbiological assessment was qualitative, the results indicate only the presence or absence of the selected bacterial species and do not provide information regarding bacterial load or relative abundance. Furthermore, the restricted pathogen panel does not comprehensively represent the subgingival microbiome. Procedural reproducibility was supported by sampling the same periodontal sites, using the same trained operator, and applying identical collection, storage, amplification, and laboratory-processing procedures throughout the study.

### 2.8. Sample Size Calculation

Since all the studies available in the literature present a split-mouth design, recent ones [33,34] were chosen, assuming PPD as the primary response variable. Sample size was calculated based on the reduction in PPD at 90 days. A clinically relevant difference of 1 mm between groups was assumed, with a standard deviation of 0.5 mm. This value also refers to a clinically relevant and appreciable value that can be detected using the periodontal probe. Being the study split-mouth, with a two-sided significance level of 0.05 and a statistical power of 80%, a minimum of 30 sites per group were obtained with a paired t-test. To compensate for potential dropouts, 40 sites per group in 40 patients were considered, for a total of 80 implant sites ultimately enrolled. The sample size calculation was performed using Stata v.13.0 (StataCorp, College Station, TX, USA).

### 2.9. Statistical Analysis

Data were analyzed using the software Stata v.13.0 (StataCorp, College Station, TX, USA). The Shapiro–Wilk test was used to assess the deviation from normal data distribution. Continuous variables with a normal distribution were expressed as mean ± standard deviation, while non-normally distributed variables were presented as medians with interquartile ranges (iqr). Qualitative data were reported as frequencies and percentages.

Considering the split-mouth design, within-group comparisons and between-group comparisons were conducted using a paired t-test or Wilcoxon matched-pairs signed-rank test, as appropriate.

One participant did not attend the scheduled 90-day follow-up visit and could not be contacted thereafter. Consequently, the corresponding paired test and control observations were unavailable at T3. No imputation of missing data was performed, and the analyses were conducted using the available paired observations.

Given the normal distribution of clinical outcomes of PPD, REC and CAL, confidence intervals were provided for variations between T0 and T3 Δ(T0–T3). In addition, effect sizes were provided through Cohen’s d_av_ [35] for comparison of Δ(T0–T3) between the aPDT group and the control group. Given the non-parametric distribution of clinical outcomes of BOP and PI, effect sizes for these outcomes were not provided, as a proper Cohen’s d_av_ format is not retrievable in this case.

Analyses according to tooth type and smoking status were considered exploratory. The study was not specifically powered for these subgroup comparisons, and no adjustment for multiple testing was applied. Therefore, the corresponding results should be interpreted as hypothesis-generating.

A *p*-value < 0.05 was considered statistically significant.

## 3. Results

### 3.1. Study Population and Sample Characteristics

An initial screening was conducted on 150 patients. A total of 40 patients diagnosed with stage II–III periodontitis were enrolled between May and November 2024.

After the screening phase, 40 patients were included in the study and randomized according to the split-mouth design (40 sites for each group). The study population consisted of 24 females (60.0%) and 16 males (40.0%), with a mean age of 52.1 ± 8.7 years. Regarding smoking habit, 27 patients (67.5%) were non-smokers and 13 (32.5%) were smokers. According to the American Society of Anesthesiologists (ASA) classification, 28 patients (70.0%) were classified as ASA I, 10 (25.0%) as ASA II, and 2 (5.0%) as ASA III.

The patients enrolled presented a diagnosis of stage II or III periodontitis (grades A to C): stage II in 25 cases and stage III in 15 cases; grade A in 27, grade B in 8, and grade C in 5 cases.

A total of 158 samples (40 samples collected at T0 for each group and 39 samples collected at T3 for each group, as one patient did not attend the final follow-up) were finally analyzed at T0 (baseline) and at T3 (90 days) both for clinical and microbiological outcomes. Microbiological samples were also performed, in addition, at T2 (14 days).

**Figure 1:**
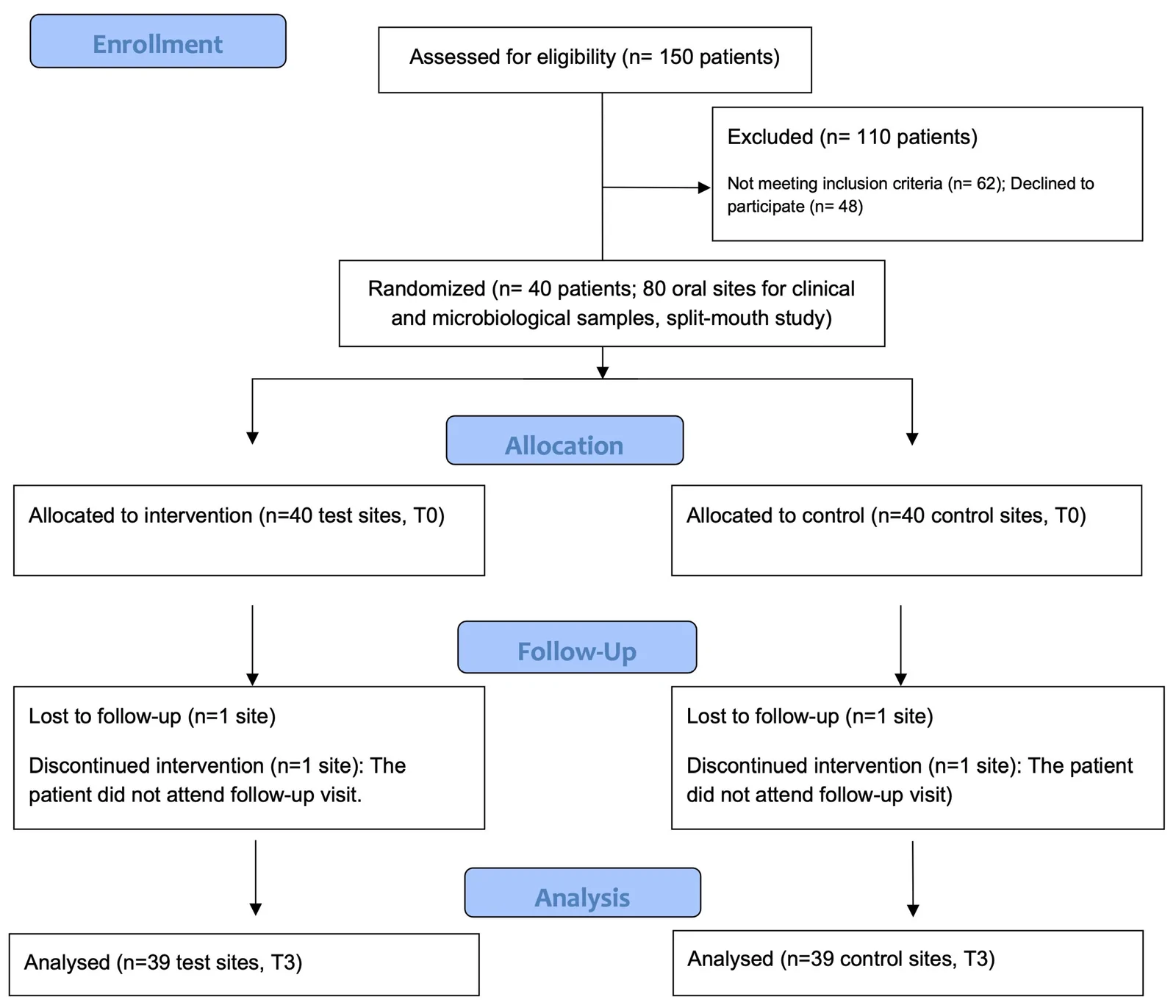
Consort flow diagram of the progress through the phases of the randomized trial.

Figure 1 reports the consort flow diagram, while Table 2 reports patients’ characteristics at T0.

### 3.2. Overall Clinical Outcomes

Both treatment protocols were associated with clinical improvements over time.

Table 3 reports clinical outcomes for PPD, REC and CAL at T0 and at T3, together with variations between T0 and T3, both for the aPDT group and control group.

For PPD, as reported in Table 3:

- the aPDT group showed a statistically significant reduction (*p* = 0.04 *) from T0 (5.33 mm) to T3 (4.08 mm);
- from T0 to T3, the reduction in PPD was greater for the aPDT group (1.25 mm) compared to the control group (1.08 mm);
- the comparison of Δ(T0–T3) between aPDT group and control group was statistically significant (paired t-test: t = 7.49; *p* < 0.001), with Cohen’s d_av_ of 0.14, which corresponds to a small effect size.

For REC, as reported in Table 3:

- from T0 to T3, the variation in REC was lower for the aPDT group (0.08 mm) compared to the control group (0.42 mm);
- the comparison of Δ(T0–T3) between aPDT group and control group was statistically significant (paired t-test: t = 5.27; *p* < 0.001), with Cohen’s d_av_ of 0.48, which corresponds to a small effect size.

For CAL, as reported in Table 3:

- from T0 to T3, the variation in CAL was greater for the aPDT group (1.17 mm) compared to the control group (0.67 mm);
- the comparison of Δ(T0–T3) between aPDT group and control group was statistically significant (paired t-test: t = 2.91; *p* < 0.001), with Cohen’s d_av_ of 0.33, which corresponds to a small effect size.

Table 4 reports clinical outcomes for BOP and PI at T0 and at T3, together with variations between T0 and T3, both for the aPDT group and the control group.

For BOP, as reported in Table 4:

- the aPDT group showed a statistically significant reduction (*p* = 0.03 *) from T0 (92%) to T3 (50%);
- from T0 to T3, the reduction in BOP was greater for the aPDT group (42%) compared to the control group (14%);
- the comparison of Δ(T0–T3) between aPDT group and control group was not statistically significant (Wilcoxon signed-rank test: z = 1.36; *p* = 0.17).

For PI, as reported in Table 4:

- the aPDT group showed a statistically significant reduction (*p* = 0.005 *) from T0 (1.07) to T3 (0.14);
- the control group showed a statistically significant reduction (*p* = 0.01 *) from T0 (0.92) to T3 (0.28);
- from T0 to T3, the reduction in PI was greater for the aPDT group (0.92) compared to the control group (0.64);
- the comparison of Δ(T0–T3) between aPDT group and control group was not statistically significant (Wilcoxon signed-rank test: z = 0.77; *p* = 0.43).

### 3.3. Clinical Outcomes According to Tooth Type

Exploratory subgroup analyses were performed according to tooth type. These analyses were not prespecified as primary outcomes, were not supported by a dedicated sample size calculation, and were conducted without adjustment for multiple comparisons. (see Table 5). For PPD, a statistically significant reduction between baseline and 90 days was observed only in single-rooted teeth treated with adjunctive aPDT, with a mean decrease of 1.17 mm (*p* = 0.04). In the same group, the reduction in PPD was significantly greater in single-rooted than in multi-rooted teeth (1.17 mm vs. 0.87 mm; *p* = 0.01). No significant between-group differences were detected for PPD when comparing control and test sites within the same tooth category.

For BOP, significant intragroup reductions were observed in the aPDT group for both single-rooted and multi-rooted teeth, whereas in the control group a significant reduction was detected only in multi-rooted teeth. In contrast, single-rooted teeth in the control group showed an increase in BOP over time. Comparison of BOP changes between single-rooted and multi-rooted teeth showed significant differences in both groups, favouring multi-rooted teeth, with a greater reduction observed in multi-rooted than in single-rooted sites.

For PI, a statistically significant reduction was observed only in single-rooted teeth treated with adjunctive aPDT (*p* = 0.02). In this subgroup, the reduction was also significantly greater than that observed in the corresponding control sites (*p* = 0.01). In addition, within the aPDT group, the reduction in PI was significantly greater in single-rooted than in multi-rooted teeth (*p* = 0.01). Overall, these exploratory analyses suggest a more favourable response to adjunctive aPDT in single-rooted teeth for PPD and PI, while BOP reduction appeared more pronounced in multi-rooted teeth.

### 3.4. Clinical Outcomes According to Smoking Status

Exploratory subgroup analyses were also performed according to smoking status. These findings should be interpreted cautiously because the study was not powered for subgroup comparisons and no adjustment for multiple testing was applied. (see Table 6). For PPD, statistically significant reductions from baseline to 90 days were observed in non-smokers in both the control and aPDT groups. The reduction was slightly greater in the aPDT group than in the control group (1.38 mm vs. 1.30 mm), although no significant between-group difference was detected. In contrast, smokers showed smaller PPD reductions, and the change did not reach statistical significance in either treatment group. When comparing smokers and non-smokers within each treatment arm, non-smokers showed significantly greater PPD improvement than smokers.

For BOP, significant reductions were observed in non-smokers in both treatment groups, with a larger decrease in the aPDT group than in the control group. Among smokers, a significant reduction in BOP was observed only in the aPDT group, and the difference between test and control sites was statistically significant. In addition, comparison between smokers and non-smokers within the control group showed a significantly more favourable BOP reduction in non-smokers.

For PI, a statistically significant reduction was observed in smokers treated with adjunctive aPDT. No other subgroup comparisons reached statistical significance. Taken together, these findings suggest that smoking habit may influence the clinical response to treatment, with non-smokers showing a more favourable trend for PPD reduction, while aPDT appeared to provide a more evident effect on inflammatory control, particularly for BOP, even in smokers.

### 3.5. Clinical Case

**Figure 2:**
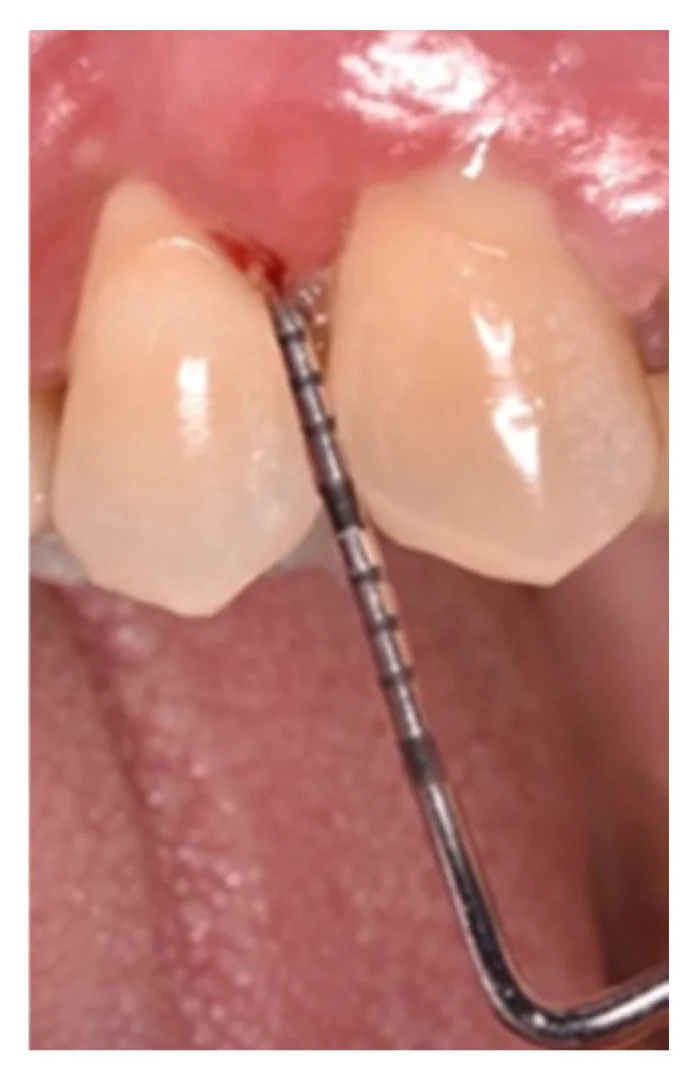
Clinical probing of 1.4 site at T0: inflammation and bleeding are visible.

**Figure 3:**
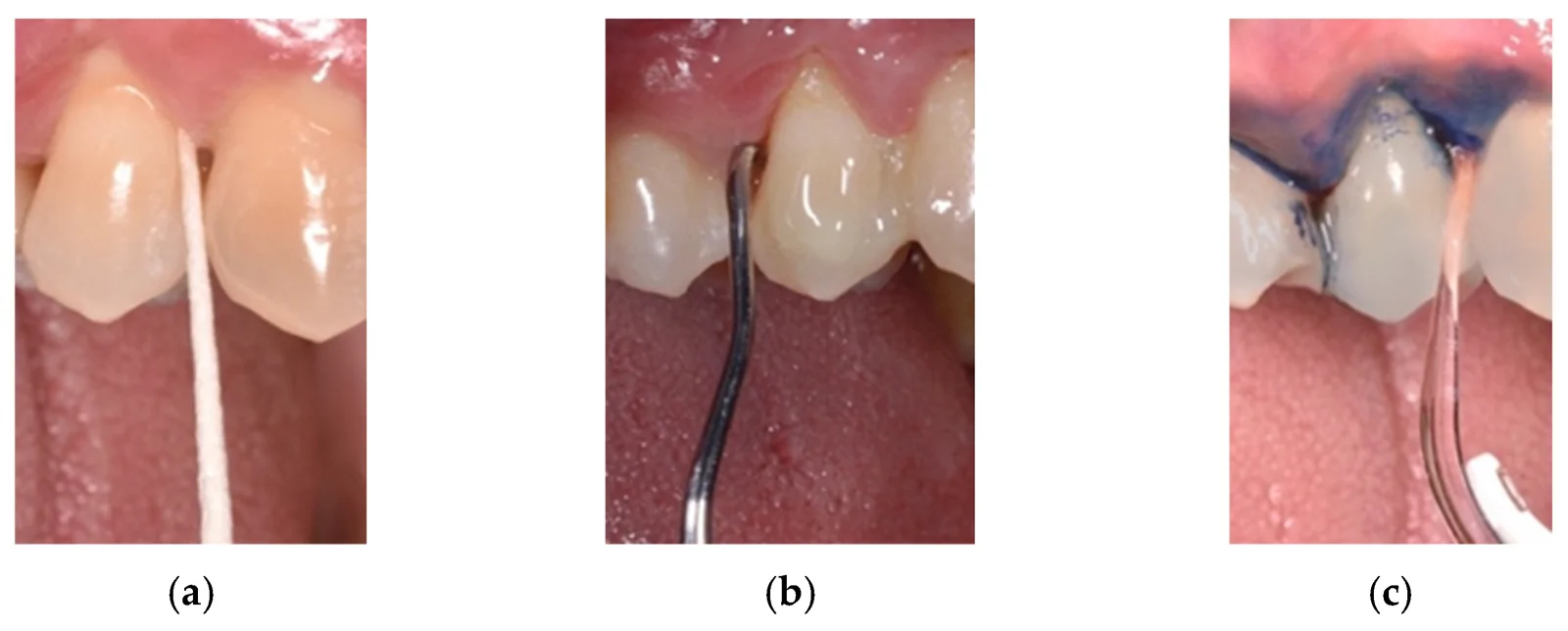
Microbiological sample of the test site at T0 using paper cone (**a**), followed by calculus removal (**b**) and aPDT therapy (**c**) in the test site.

**Figure 4:**
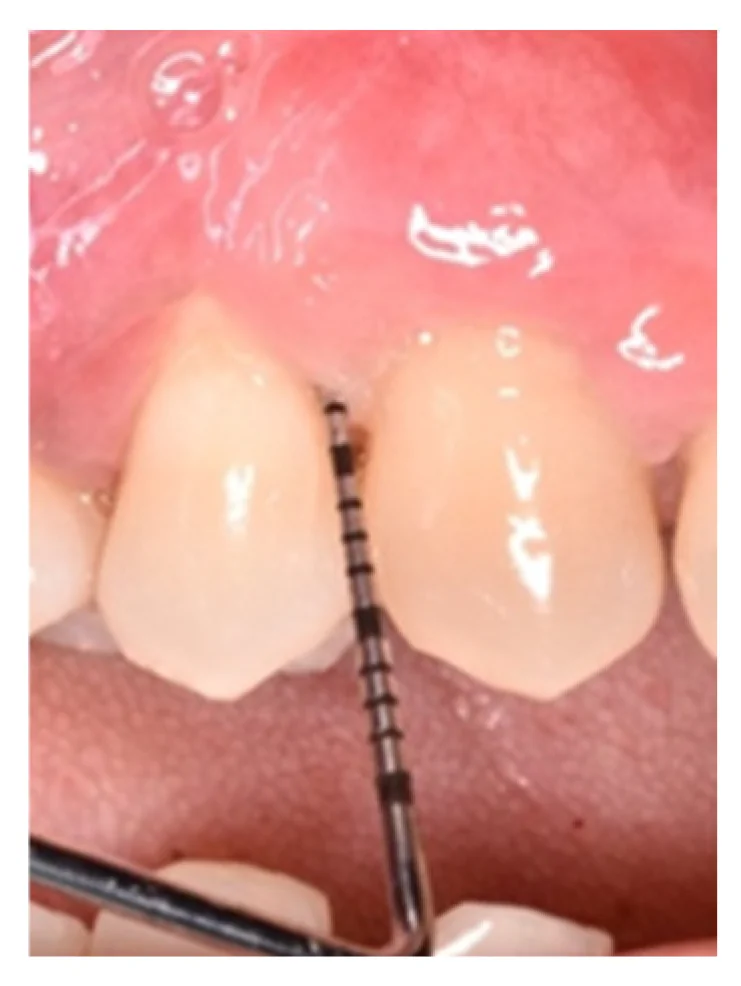
Clinical probing of 1.4 site at T3: no inflammation and bleeding are present.

A clinical case of treatment of 1.4 site with aPDT is reported in Figure 2 and Figure 3a–c and Figure 4.

### 3.6. Microbiological Outcomes

**Figure 5:**
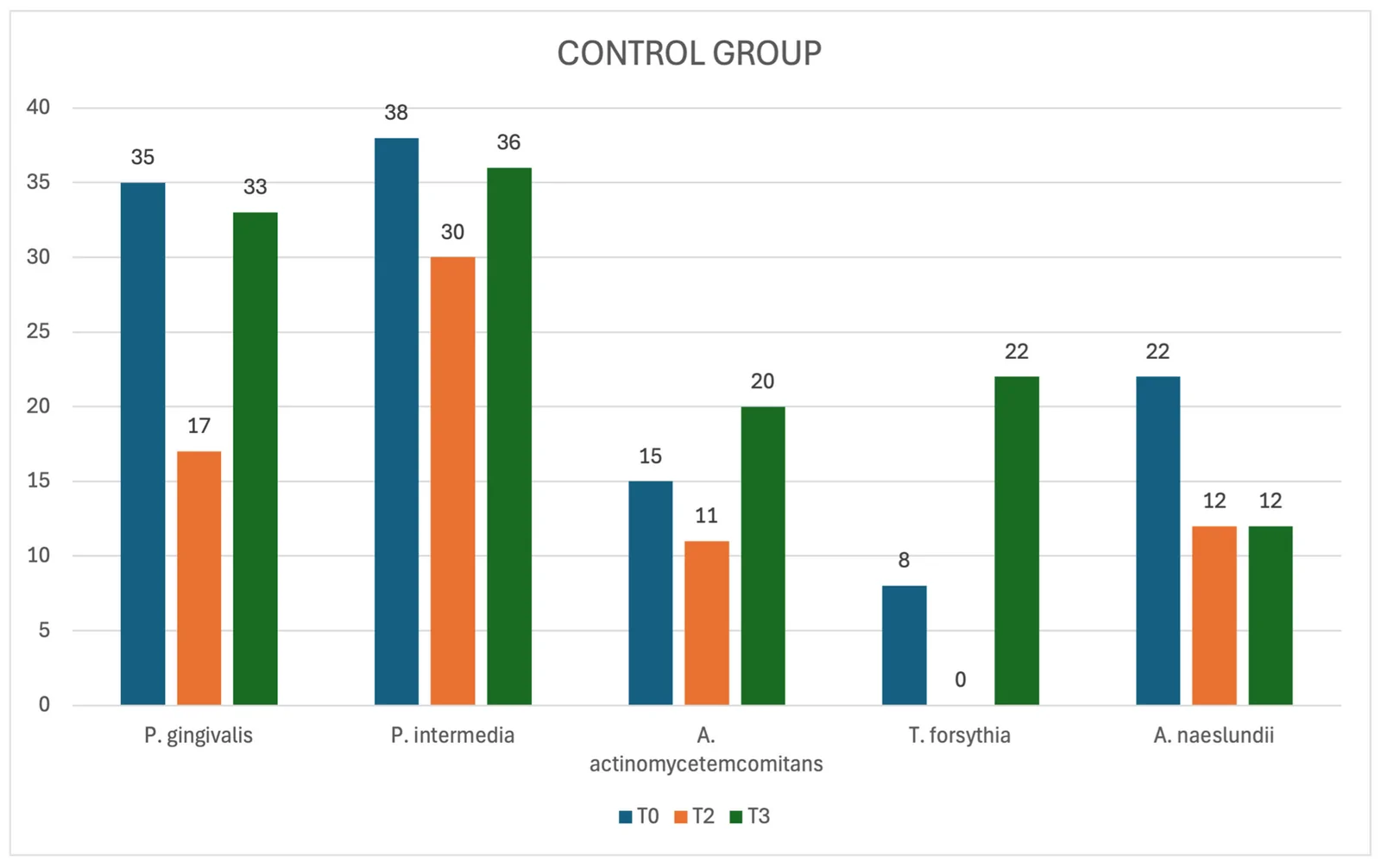
Number of sites positive for periodontal pathogens in the control group at different time points.

**Figure 6:**
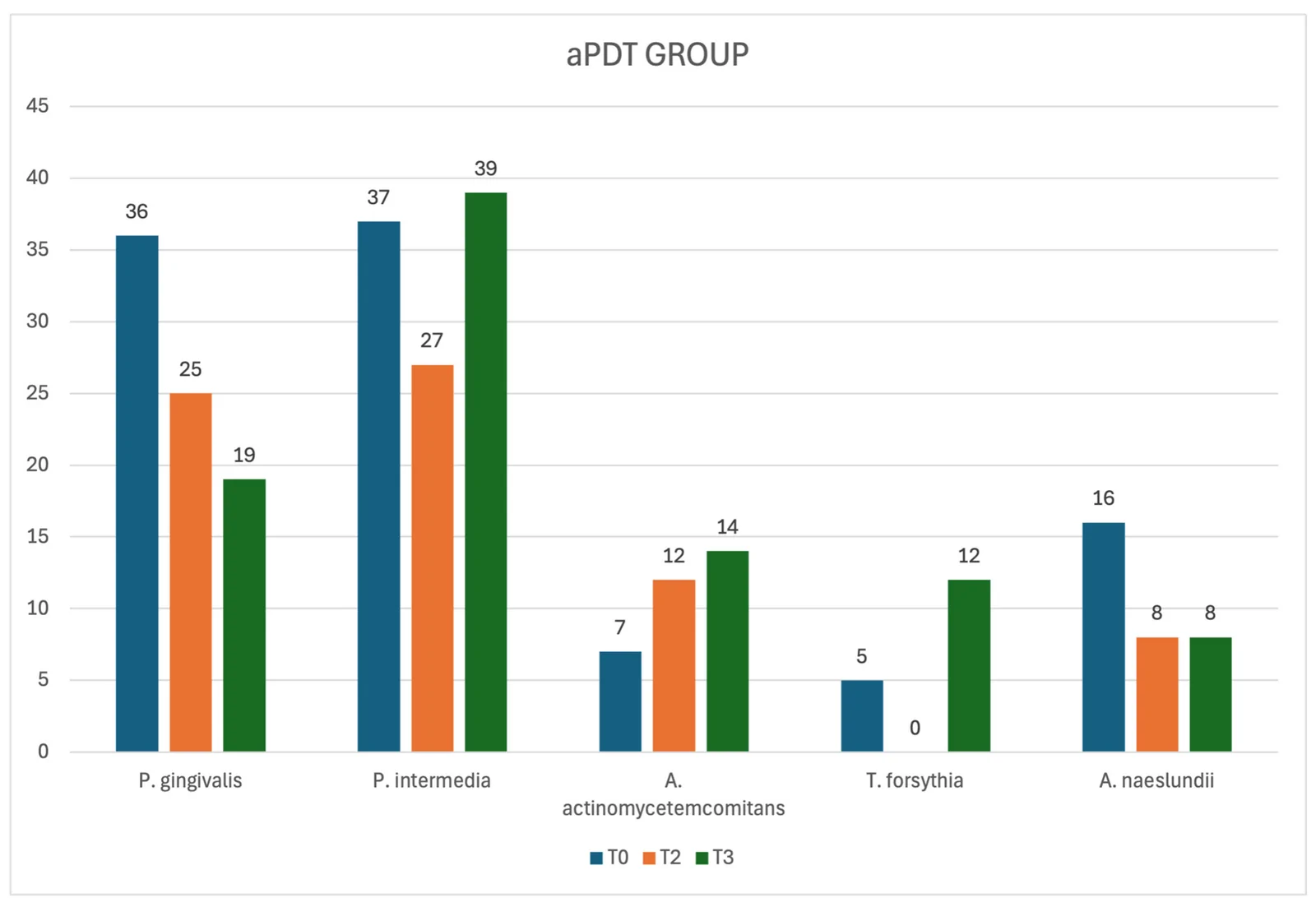
Number of sites positive for periodontal pathogens in the aPDT group at different time points.

Microbiological analysis was performed using qualitative multiplex PCR, and results are expressed as the number of sites positive for each periodontal pathogen at the different time points. As the assay provided only qualitative information, the findings reflect the presence or absence of the selected bacterial species and do not allow assessment of bacterial load or the magnitude of microbiological changes over time. In both groups, the number of sites testing positive for periodontal pathogens decreased from T0 to T2, followed by partial recolonization at T3. However, no statistically significant differences between the control and aPDT groups were detected at any evaluation time point (see Figure 5 and Figure 6).

## 4. Discussion

The present randomized split-mouth clinical trial aimed to evaluate the adjunctive effect of antimicrobial photodynamic therapy (aPDT) in non-surgical periodontal treatment. Despite adjunctive aPDT being associated with significant intra-group improvements in selected clinical parameters between T0 and T3, no additional microbiological benefit was detected compared with conventional non-surgical periodontal therapy alone. These findings suggest that, under the conditions of the present protocol, adjunctive aPDT did not provide a clear short-term advantage over mechanical debridement alone, while still showing some potentially relevant effects on local inflammation.

In addition, only five periodontal pathogens were investigated, and the rationale for selecting these microorganisms while excluding other clinically relevant species is not discussed. The selected bacteria, in different ways, represent clear markers of periodontal disease.

*A. actinomycetemcomitans* is primarily associated with aggressive, juvenile (often localized) forms. *P. intermedia* is a major cause of gingivitis and periodontal disease and is a member of Socransky’s “orange complex.” *P. gingivalis* is an obligate anaerobe and a key member of Socransky’s “red complex.” It is closely related to adult chronic periodontitis and is considered a key pathogen, being particularly active in the early and middle stages of the inflammatory disease. It frequently enters the bloodstream and has also been studied for its possible association with systemic diseases. *T. forsythia*, also a member of the red complex group, is often responsible for advanced periodontitis. *A. naeslundii* is a bacterium normally present in the oral cavity, but it appears to play a crucial role in periodontitis: it facilitates the adhesion of more aggressive pathogens (e.g., *P.gingivalis*) and its peptidoglycan triggers inflammation and bone resorption [36,37].

From a clinical point of view, both treatment protocols were associated with clinical improvements over time.

Significant greater variations were observed at T3 in the aPDT group for PPD (*p* = 0.04), BOP (*p* = 0.03) and PI (*p* = 0.005), whereas the control group showed a significant reduction only for PI (*p* = 0.01). Furthermore, from T0 to T3, the reduction in PPD was greater for the aPDT group (1.25 mm) compared to the control group (1.08 mm); the reduction in BOP was greater for the aPDT group (42%) compared to the control group (14%); the reduction in PI was greater for the aPDT group (0.92) compared to the control group (0.64).

Among these parameters, the reduction in BOP was more pronounced in the aPDT group, with a statistically significant reduction (*p* = 0.03 *) from T0 (92%) to T3 (50%), suggesting a potential beneficial effect of adjunctive aPDT in contrasting periodontal inflammation. As BOP is widely recognized as a marker of periodontal inflammatory activity and tissue response to treatment, this finding may be considered as clinically relevant. Nevertheless, because no statistically significant differences were observed between the aPDT group and control group for comparison of Δ (T0–T3), these results should be interpreted with caution and cannot be regarded as conclusive evidence of the superiority of adjunctive aPDT.

Regarding comparisons of Δ (T0–T3) between the aPDT group and the control group, the paired t-test were significant for PPD, REC and CAL (*p* < 0.001). It was thus necessary to assess whether these significant variations represented a perceptible and relevant improvement: the small effect sizes Cohen’s d_av_ < 0.5 found for all three parameters suggest differences between groups which, despite significant, cannot be defined as strongly appreciable from a clinical perspective. As the intervention under study showed a limited impact on clinical practice, authors underline that a small effect does not necessarily mean that the result is useless, but rather that its magnitude is quantitatively low and with limited novelty on clinical impact, requiring thus analysis with larger samples.

Therefore, our results do not demonstrate a clear additional clinical advantage of adjunctive aPDT over conventional non-surgical periodontal therapy. Rather, they contribute further evidence to a field in which the overall effectiveness of adjunctive aPDT remains under investigation, highlighting the need for adequately powered randomized clinical trials employing standardized treatment protocols, quantitative microbiological analyses, and longer follow-up periods to better define the clinical indications for this adjunctive approach.

The reduction in PPD observed in the test group is in line with previous studies reporting beneficial effects of aPDT when combined with scaling and root planing [33].

The potential tissue-healing effects of aPDT have also been investigated in other oral surgical settings. In a randomized clinical study evaluating mandibular third-molar extraction sockets, adjunctive aPDT was associated with reduced postoperative pain and swelling and favourable soft-tissue healing, although no significant short-term effect on bone healing was detected [38].

However, the available evidence regarding the adjunctive use of aPDT in non-surgical treatment of periodontitis still remains heterogeneous. While several randomized clinical trials have reported additional improvements in selected clinical parameters following adjunctive aPDT, other studies have failed to demonstrate significant benefits over scaling and root planing alone. This variability is reflected in recent evidence syntheses: a comprehensive systematic review by Jervøe-Storm et al. [39] concluded that the current evidence remains insufficient to support routine use of adjunctive aPDT in periodontal therapy for considerable clinical and methodological heterogeneity. Similarly, recent systematic reviews and meta-analyses have reported inconsistent findings, with some suggesting modest improvements in PPD and BOP, whereas others concluded that the magnitude and clinical relevance of these effects remain uncertain, particularly over medium- and long-term follow-up [40,41].

Plus, our recent evidence syntheses found that adjunctive aPDT may provide short-term clinical benefits in selected patient populations; however, the overall certainty of the evidence was low to moderate because of substantial heterogeneity among studies, differences in laser parameters and photosensitizers, treatment protocols, microbiological assessment methods, and follow-up duration [8,19]. Likewise, the meta-analysis by Xue et al. [25] reported improvements in some clinical parameters, but emphasized the variability of the available evidence and the need for better-designed randomized clinical trials before definitive conclusions can be drawn.

One possible explanation for the variability of the available evidence lies in the marked heterogeneity of aPDT protocols used across studies. Parameters such as wavelength, irradiation time, power output, energy delivery, photosensitizer type, pre-irradiation time, and number of treatment applications vary considerably and may significantly influence clinical outcomes [15,16,17,18]. In addition, differences in study design, severity of disease, site selection, and follow-up duration make direct comparison among studies difficult. As a consequence, the absence of a standardized protocol remains one of the major limitations in defining the true clinical value of aPDT in periodontal therapy.

The exploratory subgroup analyses performed in the present study provide additional elements for the interpretation of the clinical findings. When outcomes were analyzed according to tooth type, single-rooted teeth in the aPDT group showed a more favourable trend for PPD and PI reduction compared with multi-rooted teeth. This finding may be explained by the more favourable anatomical conditions of single-rooted teeth, in which both mechanical instrumentation and light-assisted adjunctive therapy can likely be delivered more effectively. In contrast, multi-rooted teeth are characterized by greater anatomical complexity, including root concavities and furcation involvement, which may reduce the efficacy of both subgingival debridement and adjunctive photodynamic treatment. In this regard, previous studies have suggested that the effectiveness of adjunctive aPDT may be influenced by tooth-related factors and local site characteristics, with more favourable outcomes in selected clinical conditions and a possible impact of furcation involvement on treatment response [34,42]. Interestingly, in the present study, BOP reduction in the aPDT group was observed in both single- and multi-rooted teeth and appeared more pronounced in multi-rooted sites, suggesting that the inflammatory response may behave differently from PPD changes in anatomically complex areas. Although these observations should be interpreted cautiously, they support the hypothesis that tooth morphology may influence the clinical response to adjunctive aPDT and should be considered in future studies.

Smoking status was also explored as a potential factor influencing treatment response. In the present study, non-smokers showed a more favourable trend in PPD reduction than smokers in both treatment groups, consistent with previous evidence identifying smoking as an important modifier of periodontal treatment outcomes. Previous studies have shown that smokers generally exhibit a less favourable clinical response to both non-surgical and surgical periodontal therapy and are at greater risk of disease recurrence during supportive periodontal care [40]. Furthermore, smokers have been reported to experience more rapid recolonization by Gram-negative bacteria following treatment, which may contribute to their less favourable clinical outcomes [43]. Although a significant reduction in BOP was observed among smokers receiving adjunctive aPDT, we underline that this finding derives from a limited sample size and should therefore be interpreted with caution. These observations should thus be considered preliminary and warrant confirmation in adequately powered studies specifically designed to evaluate the influence of smoking status on the response to adjunctive aPDT.

Overall, these subgroup findings should be considered exploratory, yet they add a potentially meaningful clinical perspective to the interpretation of the present results. Rather than suggesting a generalized adjunctive benefit of aPDT, they raise the possibility that its effect may vary according to local anatomical complexity and patient-related factors. This aspect may partly contribute to the heterogeneity reported in the literature and further supports the need for more standardized protocols and better stratified clinical investigations [34,42,44].

With regard to plaque control, PI significantly decreased over time, particularly in the aPDT group, but improvements were also observed in control sites. This finding confirms that mechanical debridement and reinforcement of oral hygiene remain the primary determinants of plaque reduction. Therefore, the contribution of adjunctive therapies should be interpreted within the broader framework of effective biofilm control, which remains the cornerstone of non-surgical periodontal treatment.

From a microbiological perspective, both treatment protocols were associated with a reduction in the number of sites positive for periodontal pathogens at 14 days, followed by partial recolonization at 90 days. No significant differences between groups were detected at any evaluation time point. Since the microbiological assessment was based on qualitative multiplex PCR, these findings reflect only the presence or absence of selected periodontal pathogens and do not provide information on bacterial load or the magnitude of microbiological changes. Therefore, the observed reduction and subsequent recolonization should be interpreted cautiously. Within the limitations of the qualitative assessment, the present results do not support an additional microbiological benefit of adjunctive aPDT compared with mechanical therapy alone.

It should also be emphasized that microbiological analysis in the present study was based on qualitative multiplex PCR, allowing the detection of the presence or absence of selected periodontal pathogens rather than their quantitative load. Although this method remains clinically useful and widely employed for targeted pathogen detection, it may not be sensitive enough to identify subtle changes in bacterial burden or shifts in the overall microbial community. More advanced methods, such as quantitative PCR or 16S rRNA gene sequencing, could provide a more detailed characterization of microbiological changes after treatment. However, such approaches are associated with greater costs and technical complexity, which may limit their routine application in clinical trials.

### Study Limitations

The present study, however, provides short-term data on the adjunctive use of aPDT in non-surgical periodontal therapy and offers additional insight into the possible influence of tooth-related and patient-related factors on treatment response.

However, several limitations should be considered when interpreting the findings:

- The sample size was limited to 40 patients.
- The subgroup analyses were exploratory and suggested possible differences according to tooth type and smoking status. However, these findings were based on limited sample sizes and should be considered preliminary until confirmed by adequately powered studies.
- -From a microbiological perspective, both treatment protocols showed a similar pattern, with an initial reduction in the detection of periodontal pathogens followed by partial recolonization over time. No significant differences between groups were observed, and the qualitative nature of the microbiological assessment limits the ability to further explore the relationship between microbiological and clinical outcomes. The qualitative multiplex PCR did not provide quantitative or broader microbiome-level information.
- -The split-mouth design, for the correlated observations within the same patient, required adjusted statistical methods considering variability and bias.

## 5. Conclusions

Within the limitations of this randomized split-mouth clinical trial, both conventional non-surgical periodontal therapy and adjunctive antimicrobial photodynamic therapy (aPDT) resulted in clinical improvements of periodontal parameters over time.

Although adjunctive aPDT seems to offer predictable clinical outcomes in terms of PPD and BOP reductions, further larger and stratified studies with standardized treatment protocols are needed to verify if this treatment can provide superior benefits compared with conventional non-surgical periodontal therapy.

## Figures and Tables

**Table 1:** Primers and PCR conditions used in the study.

Type	Target	Sequence	Amplicon Size bp	Annealing Temp	PCR
Forward	16 s rRNA	5′CGTGCCAGCAGCCGCGGTAATACG3′		70 °C	m1PCR
Reverse	*P. intermedia*	5′TCCGCATACGTTGCGTGCACTCAAG 3′	163		
Reverse	*A. actinomycetemcomitans*	5′CTTTGCACATCAGCGTCAGTACATCCCCAAGG 3′	253		
Reverse	*P. gingivalis*	5′TACATAGAAGCCCCGAAGGAAGACG 3′	527		
Forward	*A. naeslundii*	5′ GGA ATG ATG GCG TGA ATG GC 3′	702	60 °C	m2PCR
Reverse	*A. naeslundii*	5′ CCG ATC CCG TGA GTA CAT GG 3′			
Forward	*T. forsythia*	5′ CGG TGG TCT CCA ATC TCA CC 3′	559		
Reverse	*T. forsythia*	5′ GCC CTC AAC ACA CGA CAC TT 3′			

**Table 2:** Patient characteristics at T0 (baseline); statistically significant differences (*p* < 0.05) between frequencies are reported as *.

Variable			*p* Value
	n	%	
Sex			
male	16	40	0.09
female	24	60	
ASA Status			
1	28	70	
2	10	25	0.01 *
3	2	5	
Smoking habit			
no	27	67.5	0.08
yes	13	32.5	
Tooth type			
single rooted	11	27.5	0.03 *
multi rooted	29	72.5	
Periodontal status			
Stage II grade A	20	50	
Stage II grade B	5	12.5	0.01 *
Stage III grade A	7	17.5	
Stage III grade B	3	7.5	
Stage III grade C	5	12.5	

**Table 3:** Changes in clinical parameters of PPD, REC and CAL between baseline (T0) and 90 days (T3). Values are expressed as mean ± standard deviation (sd). PPD: probing pocket depth; REC: gingival recession; CAL: clinical attachment level. For Δ(T0–T3), which indicates the variation between T0 and T3; mean value is reported with confidence interval [C.I.]. Statistically significant differences (*p* < 0.05) between T0 and T3 are reported as *.

Outcome	Group	T0 (Mean ± SD)	T3 (Mean ± SD)	Δ (T0–T3) (Mean [C.I.])	*p*-Value
PPD (mm)	Control	5.42 ± 0.67	4.33 ± 0.78	1.08 [0.76; 1.41]	0.30
	aPDT	5.33 ± 0.65	4.08 ± 1.38	1.25 [0.76; 1.73]	0.04 *
REC (mm)	Control	0.25 ± 0.62	0.67 ± 0.98	0.42 [−0.78; −0.05]	0.40
	aPDT	0.25 ± 0.87	0.33 ± 0.65	0.08 [−0.42; 0.26]	0.60
CAL (mm)	Control	5.67 ± 0.78	5.00 ± 1.21	0.67 [0.61; 1.70]	0.18
	aPDT	5.58 ± 1.00	4.42 ± 1.38	1.17 [0.76; 1.41]	0.15

**Table 4:** Changes in clinical parameters of BOP and PI between baseline (T0) and 90 days (T3). Values are expressed as median (iqr, interquartile range). BOP: bleeding on probing; PI: plaque index. Statistically significant differences (*p* < 0.05) between T0 and T3 are reported as *.

Outcome	Group	T0 (Median (iqr))	T3 (Median (iqr))	Δ (T0–T3) (Median (iqr))	*p*-Value
BOP (%)	Control	71 (46)	57 (51)	14 (53)	0.31
	aPDT	92 (26)	50 (52)	42 (64)	0.03 *
PI	Control	0.92 (1.14)	0.28 (0.72)	0.64 (0.92)	0.01 *
	aPDT	1.07 (1.2)	0.14 (0.53)	0.92 (1.07)	0.005 *

**Table 5:** Changes in clinical parameters of PPD, BOP and PI between baseline (T0) and 90 days (T3), according to single-rooted and multi-rooted teeth. Values are expressed as mean ± standard deviation (SD). PPD: probing pocket depth; BOP: bleeding on probing; PI: plaque index. Δ indicates the change between baseline and follow-up. * Statistically significant difference within group (*p* < 0.05).

Parameter		Group	T0 (Mean ± SD)	T3 (Mean ± SD)	Δ (T0–T3)	*p*-Value (T0 vs. T3)	*p*-Value (Control vs. aPDT)
PPD (mm)	Single-rooted	Control	5.67 ± 1.74	4.67 ± 1.82	−1 ± 0.01	0.8	0.24
		aPDT	5.5 ± 1.65	3.83 ± 1.38	−1.17 ± 0.02	0.04 *	
	Multi-rooted	Control	5.18 ± 0.18	4.5 ± 1.96	−0.68 ± 0.1	0.67	0.56
		aPDT	5.2 ± 1.15	4.33 ± 0.41	−0.87 ± 0.05	0.19	
	*p*-value Control (Single VS Multi)				0.71		
	*p*-value aPDT (Single VS Multi)				0.01 *		
BOP (%)	Single-rooted	Control	50 ± 26	67 ± 27	17 ± 11	0.04 *	0.18
		aPDT	83 ± 45	65 ± 19	−18 ± 7	0.01 *	
	Multi-rooted	Control	83 ± 23	50 ± 26	−33 ± 24	0.001 *	0.09
		aPDT	100 ± 31	52 ± 15	−48 ± 35	0.001 *	
	*p*-value Control (Single VS Multi)				0.002 *		
	*p*-value aPDT (Single VS Multi)				0.001 *		
PI	Single-rooted	Control	0.67 ± 0.26	0.33 ± 0.12	−0.34 ± 0.90	0.5	0.01 *
		aPDT	1.33 ± 0.65	0.17 ± 1.38	−1.16 ± 1.48	0.02 *	
	Multi-rooted	Control	1 ± 0.57	0.33 ± 0.25	−0.67 ± 0.90	0.09	0.11
		aPDT	0.67 ± 0.65	0.4 ± 1.38	−0.27 ± 1.48	0.14	
	*p*-value Control (Single VS Multi)				0.72		
	*p*-value aPDT (Single VS Multi)				0.01 *		

**Table 6:** Changes in clinical parameters of PPD, BOP and PI between baseline (T0) and 90 days (T3), according to smokers and non-smokers. Values are expressed as mean ± standard deviation (SD). PPD: probing pocket depth; BOP: bleeding on probing; PI: plaque index. Δ indicates the change between baseline and follow-up. * Statistically significant difference within group (*p* < 0.05).

Parameter		Group	T0 (Mean ± SD)	T3 (Mean ± SD)	Δ (T0–T3)	*p*-Value (T0 vs. T3)	*p*-Value (Control vs. aPDT)
PPD (mm)	Smokers (Sm)	Control	5.25 ± 0.92	4.8 ± 1.15	−0.45 ± 0.02	0.62	0.3
		aPDT	5.75 ± 1.7	4.6 ± 1.02	−1.15 ± 0.01	0.06	
	No-smokers (No-Sm)	Control	5.5 ± 1.17	4.2 ± 2.73	−1.3 ± 0.02	0.04 *	0.98
		aPDT	5.13 ± 1.03	3.75 ± 2.04	−1.38 ± 0.11	0.02 *	
	*p*-value Control (Sm VS No-Sm)				0.01 *		
	*p*-value aPDT (Sm VS No-Sm)				0.10 *		
BOP (%)	Smokers (Sm)	Control	75 ± 33	70 ± 42	−5 ± 2	0.34	0.01 *
		aPDT	90 ± 57	62 ± 45	−28 ± 11	0.001 *	
	No-smokers (No-Sm)	Control	63 ± 37	50 ± 25	−13 ± 8	0.01 *	0.17
		aPDT	88 ± 48	47 ± 24	−41 ± 23	0.001 *	
	*p*-value Control (Sm VS No-Sm)				0.01 *		
	*p*-value aPDT (Sm VS No-Sm)				0.5		
PI	Smokers (Sm)	Control	1.25 ± 0.56	0.5 ± 0.01	−0.75 ± 0.01	0.27	0.26
		aPDT	1.75 ± 1.02	0.45 ± 1.71	−1.3 ± 1.19	0.001 *	
	No-smokers (No-Sm)	Control	0.63 ± 0.05	0.25 ± 0.03	−0.38 ± 0.05	0.81	0.15
		aPDT	0.65 ± 0.14	0.07 ± 0.01	−0.58 ± 0.04	0.08	
	*p*-value Control (Sm VS No-Sm)				0.9		
	*p*-value aPDT (Sm VS No-Sm)				0.61		

## Data Availability

Data are available from the corresponding authors upon reasonable request because the data are not publicly available due to privacy/ethical restrictions.
